# Correlation between TMJ Space Alteration and Disc Displacement: A Retrospective CBCT and MRI Study

**DOI:** 10.3390/diagnostics14010044

**Published:** 2023-12-25

**Authors:** Wenjing Yu, Hyeran Helen Jeon, Soriul Kim, Adeyinka Dayo, Muralidhar Mupparapu, Normand S. Boucher

**Affiliations:** 1 Department of Orthodontics, School of Dental Medicine, University of Pennsylvania, 240 South 40th Street, Philadelphia, PA 19104, USA; wenjingy@upenn.edu; 2 Institute of Human Genomic Study, College of Medicine, Korea University, 145 Anam-ro, Seongbuk-gu, Seoul 02841, Republic of Korea; soriul@korea.ac.kr; 3 Department of Oral Medicine, School of Dental Medicine, University of Pennsylvania, Philadelphia, PA 19104, USA; dayoad@upenn.edu (A.D.); mmd@upenn.edu (M.M.)

**Keywords:** temporomandibular joint space, disc displacement, cone beam computed tomography (CBCT), magnetic resonance imaging (MRI)

## Abstract

This study aims to determine if a large anterior and reduced posterior/superior joint space is highly predictable for disc displacement. From patients with temporomandibular disorders symptoms, fifty-two experimental joints and fourteen control joints were included. The cone beam computed tomography (CBCT) images were used to calculate posterior-to-anterior (P-A) and superior-to-anterior (S-A) joint space ratios, while disc position was determined using magnetic resonance imaging (MRI). One-way analysis of covariance test and receiver operating characteristics analysis were carried out. The results showed that among the 52 experimental joints, 45 were diagnosed as disc displacement and 7 as normal disc positions (N). All 14 control joints showed normal disc positions. The P-A ratio was 1.46 ± 0.21, 0.99 ± 0.23, and 0.86 ± 0.30 in the control, N, and DD groups, respectively (*p* < 0.001). The S-A ratio was 1.80 ± 0.27, 1.44 ± 0.33, and 1.08 ± 0.35 in the control, N, and DD groups, respectively (*p* < 0.001). When an altered P-A ratio and/or S-A ratio are observed on the CBCT, the diagnosis of disc displacement is quite predictable with high sensitivity and specificity.

## 1. Introduction

The temporomandibular joint (TMJ) is one of the most complicated joints in the body. The articular disc separates the mandibular condyle and glenoid fossa from direct articulation and functionally serves as an unossified tissue, permitting the complex movements of the joint [1,2]. In healthy joints, the condyle articulates on the intermediate zone of the disc. Pain, joint noises, deviation of the mandible upon opening, limited opening, and a change in the occlusal relationship are common symptoms of temporomandibular disorders (TMD) [3,4]. The prevalence of TMD is approximately 40% to 60% of the general population and can occur at all ages, with a high prevalence in females aged 20 to 40 years [5]. The most common form of TMD is anterior disc displacement, aptly described as an altered relationship of the disc to the condyle [3]. Besides symptomatic relief, a fundamental question in TMD patient management is whether the disc-condyle is located in a stable position in the glenoid fossa in centric occlusion. Disc displacement can cause the condyle to change its position in the fossa, resulting in altered joint spaces. The altered position of the condyle depends on the direction and extent of the disc displacement. With non-growing orthodontic patients, if the TMJ is not stable, treatment outcomes may be unsatisfactory relative to function, esthetics, stability, and pain [6]. With growing subjects, several studies reveal that unilateral TMJ disc displacement impairs ipsilateral mandibular growth and bilateral joint involvement induces mandibular retrognathia [7,8]. Therefore, it is critical for orthodontists who treat TMD patients to include the disc position as part of the orthodontic diagnosis.

The diagnosis of TMD is based on thorough clinical examinations and advanced imaging techniques. Cone beam computed tomography (CBCT) provides noninvasive thin-slice images on axial, coronal, and sagittal planes with a significantly lower radiation dose than the conventional CT, and is considered the best method for assessing the osseous pathology of the TMJ [9,10]. Magnetic resonance imaging (MRI) has become the best imaging modality in evaluating soft tissue abnormality of the joint and surrounding regions due to its non-invasiveness and sensitivity for soft tissue evaluation. It can visualize the anatomy of the joint and the disc position and detect the early signs of TMJ dysfunction, such as band thickening, retrodiscal tissue rupture, and joint effusion [9,11].

The cost and availability of MRI remain an obstacle when considering an MRI on TMJ as part of routine orthodontic records. CBCT-derived TMJ sagittal view provides the joint space measurements and adds diagnostic value in assessing the relationship between the condyle and the glenoid fossa [12]. Previous studies reported the ratio of the anterior to superior to posterior joint spaces is 1:1.9:1.6 without significant gender difference in patients with optimal disc position. An increased anterior joint space with reduced posterior/superior joint spaces can be an important diagnostic finding, suggesting disc subluxation or complete dislocation [12,13,14].

Our study, using both MRI and CBCT images, aims to investigate the correlations between the altered TMJ space ratio and disc displacement and examine if reduced P-A and/or S-A ratio on CBCT is highly predictive for diagnosing disc displacement. Since MRI is not a routine examination for pre-orthodontic records while CBCT has been increasingly used more than ever, our findings will shed light on and facilitate diagnosing TMJ disc displacement using CBCT analysis in daily practice.

## 2. Materials and Methods

### 2.1. Study Population

This retrospective study was approved by the Institutional Review Board (IRB) at the University of Pennsylvania (Protocol # 844553). For the experimental group, fifty-three Caucasian patients with significant TMD symptoms and altered joint space ratio were initially selected from private orthodontic practice. All patients were carefully examined and categorized according to the diagnostic criteria for temporomandibular disorders (DC/TMD) [15]. Full-volume CBCT scans using an i-CAT^TM^ machine (KaVo Imaging, Hatfield, PA, USA) were taken at a centric relation position with the patient’s mouth closed in the natural head position as the routine orthodontic records from May 2013 to November 2020. These CBCT images were taken at 120 kVp and 5 mA with a volume size of 16 × 13 cm^2^, a voxel size of 0.3 mm, and an exposure time of 3.7 s. The patient was referred for an additional MRI to identify the disc location due to TMD symptoms. All the subjects had both CBCT and MRI in a short period (40.15 ± 96.25 days). The exclusion criteria were: (1) active condyle resorption; (2) tumor and neoplasia; (3) malformed condylar head (including remodeling bird beak-shaped condylar head); (4) mandibular fractures; and (5) subjects with low-quality CBCT and MRI images. Finally, we included fifty-two joints from thirty subjects for analysis (7 males and 23 females; 37.5 ± 17.5 years). TMD symptoms included jaw pain or discomfort, headaches, clicking or popping of the jaw, and earaches. In addition, for the control group, fourteen joints from seven TMD subjects were selected from the same orthodontic office (2 males and 5 females; 43.4 ± 14.3 years). The inclusion criteria for the control group were (1) to present normal joint space ratio assessed by CBCT images and (2) to have both CBCT and MRI in a short period.

We used an effect size (*f*) of 0.45 in our sample size calculations using G*Power ver. 3.1.9.2. Sample size was computed to analyze covariance with both P-A and S-A joint space ratios as outcomes and five different disc position groups (e.g., control group, normal disc position group, and three-disc displacement groups) as predictors. Statistical power (1-*β*) was set at 0.80, and the significance level (*α*) was set at 0.05. The minimum number of samples was 65.

### 2.2. CBCT Measurements

**Figure 1:**
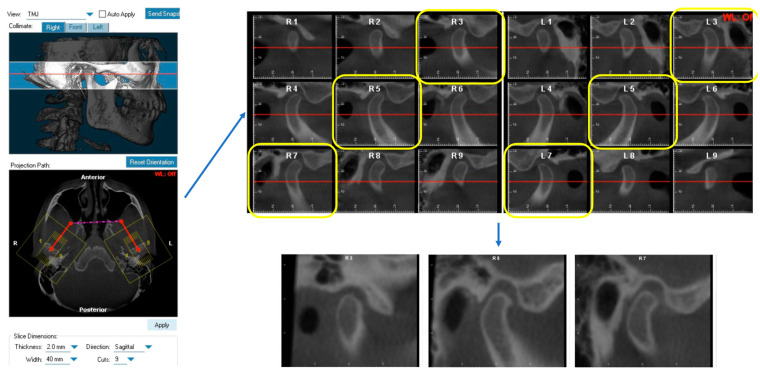
Generation of sagittal TMJ slice cuts from CBCT.

**Figure 2:**
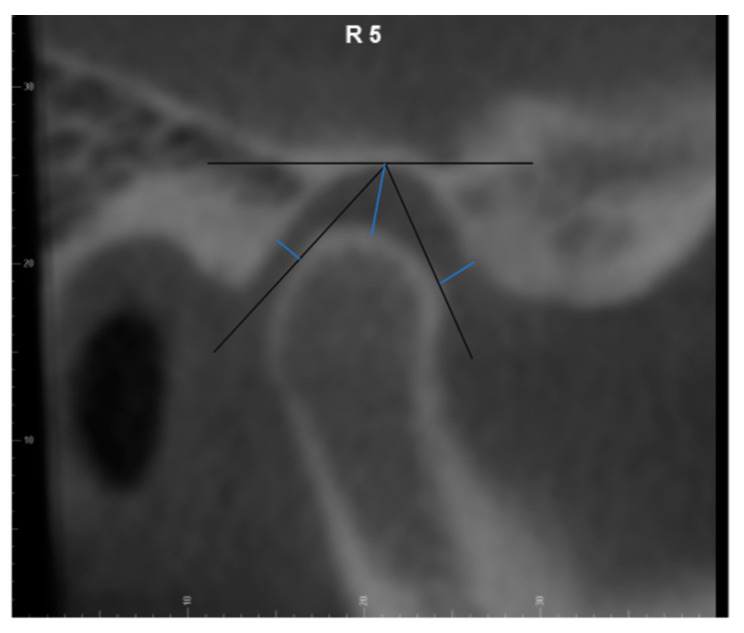
Temporomandibular joint space measurement.

All CBCT images were oriented and analyzed using the Dolphin Imaging 3D software (version 11.9, Dolphin Imaging & Management Solutions, Chatsworth, CA, USA). Each head was oriented in three planes of space for frontal and lateral views. The head was oriented in the frontal view with the floor of both orbits parallel to the floor [16]. In the right-side lateral view, the head position was adjusted so that the glabella was used to define the distance from the facial axis point of the maxillary central incisor to the goal anterior limit line (GALL) line matched to the clinical record. The lateral borders of the orbital rim, ramus borders, and mandibular angles were superimposed to the best fit from left and right. After orientation, the axial image demonstrating the largest mediolateral dimension of the condyle was selected. Then, we drew the reference line connecting the medial and lateral poles of the condyle [17]. Nine sagittal TMJ slices were made with 2.0 mm thickness for each joint. The 3rd, 5th, and 7th cuts from the right TMJ were saved as the lateral, central, and medial pole and the 3rd, 5th, and 7th cuts from the left TMJ were saved as the medial, central pole, and lateral pole, respectively (Figure 1). Three linear measurements of TMJ space, including the anterior joint space (AS), superior joint space (SS), and posterior joint space (PS), were performed on each cut as previously described [13]. Briefly, the distance from the most superior point of the condyle to the most superior aspect of the glenoid fossa was measured as the superior joint space (SS). Lines tangent to the most anterior and posterior aspects of the condyle were drawn from the most superior aspect of the glenoid fossa. The shortest distances from the anterior and posterior tangent points to the glenoid fossa were measured as the anterior joint space (AS) and posterior joint space (PS) (Figure 2).

### 2.3. MRI Evaluation

MRI scans taken in a centric relation closed-mouth position were used for evaluation. MRI scans were acquired by a 1.5 Tesla imager (Signa; General Electric, Milwaukee, WI, USA) with bilateral 3-inch TMJ surface coil receivers. The scans include the following sequences: axial-weighted localizing images followed by sagittal oblique proton-density-weighted images in the open and closed mouth positions. Sagittal oblique fat-suppressed images and T1 weighted images were also acquired, as were coronal T1 weighted images. After calibration, two experienced radiologists (A.D. and M.M.) read and diagnosed the MRI scans in the lateral, central, and medial poles. We used the scoring system demonstrating the various levels of disc displacement in the sagittal plane as previously described [18,19]. Using the clock analogy, the position of the disc posterior band margin was evaluated relative to the 12 o’clock position. The disc position was recorded as normal when the posterior band was at 12 o’clock and as anterior displacement when the posterior band was at 11 o’clock or below. The disc position in each joint was examined in 3 sagittal MRI slices (medial, central, and lateral aspects of the joint). Both radiologists agreed upon the diagnosis of the disc position for the whole joints.

### 2.4. Statistics

Statistical analysis was conducted with SAS (version 9.4; SAS institute Inc., Cary, NC, USA). Descriptive statistics were calculated to describe the mean and standard deviations of joint space ratio for each lateral, central, and medial cut. The interclass correlation coefficient (ICC) was calculated to test the reliability of the measurements. Statistical analysis among multiple groups was performed by either one-way analysis of covariance (ANCOVA) with Scheffe’s post-hoc test or non-parametric Kruskal–Wallis test with Dunn test after adjustment for age and sex. To determine the optimal cut-off values, sensitivity, and specificity, we used the receive operating characteristics (ROC) curves and area under the curve (AUC) as the measurement of accuracy. An AUC value of 1.0 indicates the perfect predictor with a 100% sensitivity and 0% false positive rate, but a value of 0.5 represents that the evaluated measurements have no discrimination. Thus, joint space ratios with an AUC closer to 1.0 indicated a better predictor for disc displacements, with an AUC of 0.70–0.79 considered acceptable, 0.80–0.89 considered excellent, and more than 0.90 considered outstanding [20]. In addition, we used the maximal values of Youden’s index [max (sensitivity + specificity − 1)] to determine the optimal cut-off points for each joint space ratio [21]. For this analysis, we used a total of 59 joints (45 joints with disc displacement in the experimental group subjects and 14 normal joints in the control group), except for 7 joints diagnosed as normal disc positions in the experimental group. *p*-value < 0.05 was considered statistically significant.

## 3. Results

### 3.1. Sample Population

A total of 52 joints in the experimental group and 14 joints in the control group were categorized into the 12 common TMDs based on the patient’s history and clinical examination, including myalgia, local myalgia, myofascial pain, myofascial pain with referral, arthralgia, headache attributed to TMD, four disc displacement disorders, degenerative joint disease, and subluxation according to the Diagnostic Criteria for Temporomandibular Disorders (DC/TMD) (Table 1). Some subjects had multiple symptoms, such as myofascial pain and disc displacement.

### 3.2. Disc Position Information

Among the 52 joints in our experimental group subjects, there are 7 joints diagnosed as normal disc positions and 45 joints with disc displacement, including 4 anterior and medial disc displacements, 11 anterior-only disc displacements, and 30 anterior and lateral disc displacements, as assessed by MRI scans (Table 2). The control group joints were presented with the condyles sitting in a centric position in the glenoid fossa assessed by CBCT images and with optimal disc position assessed by MRI.

### 3.3. Posterior-to-Anterior Joint Space Ratio (P-A Ratio)

**Figure 3:**
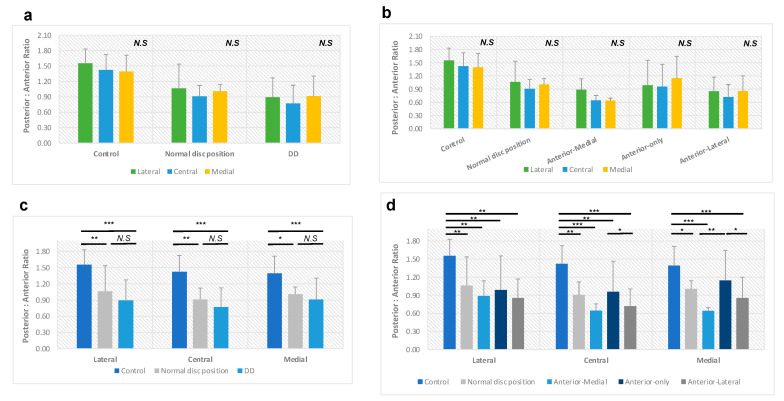
The posterior-to-anterior joint space ratio in different disc position groups. (**a**,**b**) In each group, there is no statistical significance in the P-A ratio among the lateral, central, and medial poles. (**c**) Disc displacement group has a reduced P-A ratio compared to the control group in the lateral, central, or medial poles. (**d**) Anterior-medial, anterior-only, and anterior-lateral disc displacement groups have reduced P-A ratio compared to the control group in the lateral, central, or medial poles. N.S., not statistically significant; *, *p* < 0.05; **, *p* < 0.01; ***, *p* < 0.001 after adjustment for age and sex.

In the control group, the P-A ratio is 1.46 ± 0.21 in our study, with 1.55 ± 0.28 in the lateral pole, 1.42 ± 0.30 in the center, and 1.39 ± 0.32 in the medial pole, respectively (Table 3). There is a tendency to have a larger P-A ratio in the lateral pole, gradually decreasing when moving toward the medial pole. However, there were no statistically significant differences in the P-A ratio among these three planes (*p* > 0.05) (Figure 3a,b).

In the normal disc position (N) group of our experimental group subjects, the P-A ratio decreased to 0.99 ± 0.23, with 1.06 ± 0.48 in the lateral pole, 0.91 ± 0.21 in the center, and 1.01 ± 0.13 in the medial pole, respectively (Table 3). Unlike the trend of the control group, the central plane in the normal disc position group has the smallest P-A ratio compared to the lateral and medial poles. However, no statistically significant differences were found among these three planes either (*p* > 0.05) (Figure 3a,b). Surprisingly, when compared to the control group, the drop in the P-A ratio in the N group was statistically significant in three planes separately (all *p* values < 0.05) (Figure 3c,d).

The disc displacement group has the lowest P-A ratio, 0.86 ± 0.30, with 0.89 ± 0.38 in the lateral pole, 0.77 ± 0.35 in the center, and 0.91 ± 0.39 in the medial pole, respectively (Table 3). The disc displacement group follows the same tendency as the normal disc position group in that the central plane has the smallest P-A ratio compared to the lateral and medial poles, with no statistically significant differences among these three planes (*p* > 0.05) (Figure 3a,b). Then, we compared the disc displacement group with the control in three planes separately; the drop in the P-A ratio was significant in each plane (all *p* values < 0.001) (Figure 3c,d). In addition, we noticed a slight decrease in the P-A ratio in disc displacement compared to the N group, but there was no statistical significance (*p* > 0.05, Figure 3c). If we break them down into three disc displacement types, the most significant drop in P-A ratio was in the anterior-medial disc displacement group, and the slightest drop was in the anterior-only disc displacement group (Table 3).

### 3.4. Superior-to-Anterior Joint Space Ratio (S-A Ratio)

**Figure 4:**
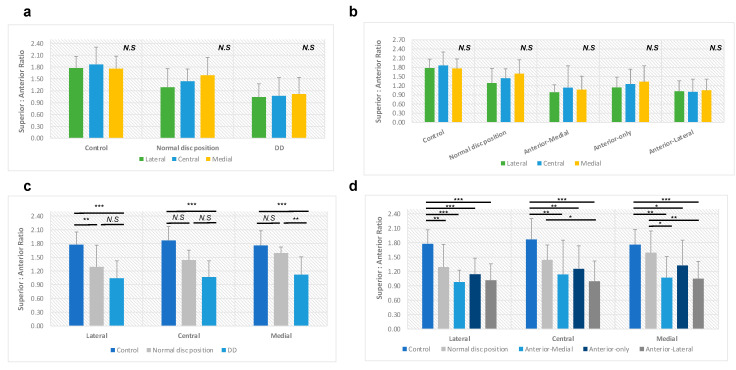
The superior to anterior joint space ratio in different disc position groups. (**a**,**b**) In each group, there is no statistical significance in the S-A ratio among the lateral, central, and medial poles. (**c**) Disc displacement group has a significantly reduced S-A ratio compared to the control group in the lateral, central, or medial poles. (**d**) Anterior-medial, anterior-only, and anterior-lateral disc displacement groups have reduced S-A ratios compared to the control group in the lateral, central, or medial poles. N.S., not statistically significant; *, *p* < 0.05; **, *p* < 0.01; ***, *p* < 0.001 after adjustment for age and sex.

We also examined the superior-to-anterior joint space ratio. In the control group, the S-A ratio is 1.80 ± 0.27, with 1.77 ± 0.29 in the lateral pole, 1.87 ± 0.44 in the central pole, and 1.76 ± 0.31 in the medial pole, respectively (Table 4). The central plane has a larger S-A ratio than the lateral and medial, with no statistically significant difference (*p* > 0.05; Figure 4a,b).

In the N group, the S-A ratio decreased to 1.44 ± 0.33, with 1.29 ± 0.47 in the lateral pole, 1.44 ± 0.31 in the center, and 1.59 ± 0.45 in the medial pole, respectively (Table 4). Different from the trend of the control group, the S-A ratio gradually increased from the lateral pole to the medial pole without any statistically significant differences among these three planes (*p* > 0.05; Figure 4a,b). Compared to the control group, the decrease in the S-A ratio in the normal disc position group was only statistically significant in the lateral pole (*p* < 0.05; Figure 4c,d).

Similar to the P-A ratio, the disc displacement group has the lowest S-A ratio, 1.08 ± 0.35, with 1.04 ± 0.33 in the lateral pole, 1.07 ± 0.47 in the center, and 1.12 ± 0.42 in the medial pole, respectively (Table 4). The disc displacement group followed the same tendency as the normal disc position group: the S-A ratio gradually increased from the lateral to the medial pole (*p* > 0.05; Figure 4a,b). When comparing the disc displacement group with the control group in three planes separately, the S-A ratio was significantly decreased in each plane (all *p* values < 0.001; Figure 4c,d). Regarding disc displacement subgroups, the most significant drop in the S-A ratio was observed in the anterior-lateral DD group, and the slightest drop was in the anterior-only disc displacement group (Table 4).

In addition, we performed the sensitivity analyses of the P-A and S-A joint space ratios using only the left or right joints (Supplementary Tables S1 and S2). Both parts demonstrated a similarly significant association between joint space ratio and disc displacement. In those sensitivity analyses, we used an effect size (*f*) of 0.55 in our sample size calculations. The sample size was computed for an ANCOVA with total P-A joint space ratio as outcomes and three different disc position groups as predictors. Statistical power (1-*β*) was set at 0.80, and the significance level (*α*) was set at 0.05. The minimum number of samples was 36.

The ICC for the repeated measurements of nine randomly picked CBCT images was 0.923, which shows the high consistency and reliability of the current measurement protocol.

### 3.5. Receiver Operating Characteristic (ROC) Curve Analysis

**Figure 5:**
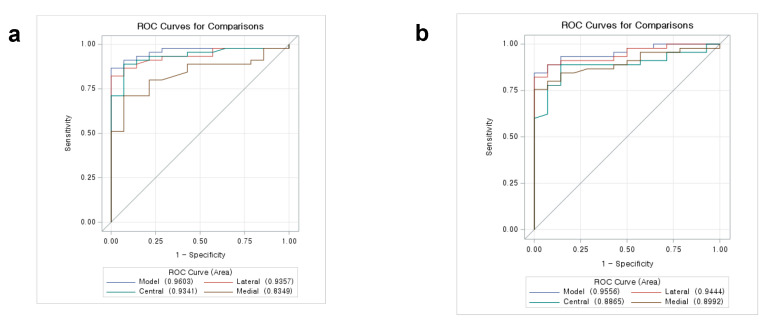
The receiver operating characteristic (ROC) curve demonstrates the association between the P-A ratio and disc displacement (**a**) and between the S-A ratio and disc displacement (**b**).

ROC curve analysis was performed for P-A ratios (Figure 5a) and S-A ratios (Figure 5b). On the evaluation of possible anterior disc displacement by P-A ratios, the AUC on ROC curve analysis were 0.94 (95% CI, 0.87–1.00) for the lateral pole, 0.93 (95% CI, 0.86–1.00) for the central pole, and 0.83 (95% CI, 0.75–0.96) for the medial pole. Furthermore, the AUC of S-A ratios were 0.94 (95% CI, 0.88–1.00) for the lateral pole, 0.89 (95% CI, 0.81–0.98) for the central pole, and 0.90 (95% CI, 0.84–0.99) for the medial pole. These data indicated that CBCT-derived P-A ratios at the lateral and central poles and S-A ratios at the lateral and medial poles are outstanding diagnostic modalities for predicting disc displacement.

The cut-off values of the P-A ratios discriminating those at possible anterior disc displacement based on Youden’s index were 1.678 at the lateral pole (sensitivity = 0.911, specificity = 0.929, Youden’s index = 0.840) and 1.339 at the central pole (sensitivity = 0.867, specificity = 0.929, Youden’s index = 0.795) (Table 5). In addition, the cut-off values for the S-A ratios discriminating those at possible anterior disc displacement based on the Youden index were 1.733 at the lateral pole (sensitivity = 0.911, specificity = 0.929, Youden’s index = 0.840) and 1.916 at the medial pole (sensitivity = 0.822, specificity = 0.929, Youden’s index = 0.751) (Table 6). In total, the cut-off values of the P-A ratios discriminating those at possible anterior disc displacement based on Youden’s index were 1.294 (sensitivity = 0.844, specificity = 1, Youden’s index = 0.844) and the cut-off values of the S-A ratios discriminating those at possible anterior disc displacement based on Youden’s index were 1.492 (sensitivity = 0.8, specificity = 1, Youden’s index = 0.8) (Table 7).

## 4. Discussion

TMDs are the most common cause of chronic pain from the non-dental origin in the orofacial area [22]. In disc displacement joints, the altered position of the disc relative to the condyle head is often referred to as an internal derangement, and the precise localization of the disc is critical in diagnosing TMJ internal derangement [3,11]. Our study found that out of 52 joints, which presented with larger than normal anterior joint spaces and reduced posterior and/or superior spaces, 45 joints (87%) had disc displacements, and 7 joints (13%) showed normal disc position. Of disc displacement joints, 67% were anterior-lateral displacement, followed by anterior-only and anterior-medial disc displacement. Our disc displacement joint subgroup characteristics are consistent with the previous studies, supporting more frequent anterolateral disc displacement and the low percentage of normal disc position in symptomatic patients [18,23].

Disc displacement can cause the condyle to change its position in the fossa, resulting in altered joint spaces. The altered position of the condyle depends on the direction and extent of the disc displacement [14]. The P-A and S-A ratios in healthy joints without any TMD symptoms, where the condyles are in a centric position, were 1.6 and 1.9, respectively [13]. The normative values of the proportionate relationship of anterior, superior, and posterior joint spaces are a valuable reference in diagnosing condylar position using CBCT-derived images. In our control group, the P-A and S-A ratios were 1.46 ± 0.21 and 1.80 ± 0.27, which was very close to the previous study investigating healthy joints. However, in our control group, all subjects had TMD symptoms. Therefore, they were not considered healthy joints even though MRI images showed no disc dislocation. Interestingly, in the normal disc position of our experimental group, the P-A and S-A ratios were 0.99 ± 0.23 and 1.44 ± 0.33, respectively, which were lower than the ones in the control group. We assume that the bone remodeling related to a disc displacement might have been initiated even though the disc is still in a centric position. In the disc displacement group, the joints showed significantly decreased P-A and S-A ratios due to enlarged anterior joint space and smaller posterior/superior space. Previous studies support the close relationship between altered joint space and disc displacement [8,12,14,24]. In disc displacement patients, an anteriorly displaced disc and superiorly and posteriorly positioned mandibular condyle are observed using MRI, showing the smaller posterior and superior joint spaces compared to the healthy volunteer groups [24]. Ikeda et al. and Gateno et al. reported a bigger anterior and smaller posterior joint space with posteriorly displaced condyles in the joints with anterior disc displacement [12,14]. The possible reasons for posteriorly positioned condyles in anterior disc displacement joints include a space limitation after disc displacement, condyle and fossa remodeling after disc displacement, and a natural posteriorly positioned condyle predisposing the joint to anterior disc displacement [8]. However, the cause-and-effect relationship between the posteriorly positioned condyle and disc displacement remains unclear.

TMJ disc position significantly affects craniofacial growth. When the disc is not correctly located between the condyle and the articular eminence of the glenoid fossa, there can be an unfavorable effect on the growth and development of the maxilla and mandible [25,26,27,28,29]. TMJ disc displacement is reported to be associated with reduced maxillary and mandibular body forward growth and reduced vertical ramus growth [23,30]. In addition, a decrease in posterior facial height with an increase in anterior facial height, steep palatal and mandibular planes, vertically reduced maxillary molar position, and retroclined mandibular incisors are also considered to be associated with TMJ disc displacement [23]. These altered skeletodental features related to DD become more evident with growth. Considering that disc displacement frequently occurs in pre-orthodontic adolescents and a significant increase in the frequency of more advanced disc displacement is found in older patients, the role of orthodontists is critical in identifying disc displacement in our daily practice. Evaluation of TMJ disc position should be considered in our pre-orthodontic treatment planning process [31,32].

As many patients with disc displacement are asymptomatic, the frequency of disc displacement is consistently underestimated. For example, approximately 30% of asymptomatic patients had disc displacement confirmed by MRI [3]. CBCT provides high-resolution multiplanar images without superimposition or distortion and uses substantially lower radiation doses than multi-slice CT. It also facilitates bone morphology and joint space analysis in all three dimensions [33]. However, CBCT cannot provide precise information related to the disc position [8]. Instead, MRI can provide superior contrast resolution and dynamic imaging to demonstrate the TMJ functions without exposure to ionizing radiation [34]. However, the cost and availability of MRI remain an obstacle when considering TMJ MRI as part of the pre-orthodontic assessments [23]. Given that CBCT imaging is a common diagnostic record in many orthodontic practices, we can screen possible TMD patients using our CBCT records, based on the high sensitivity and specificity of altered joint space ratio and disc displacement. For example, if the patient presents less than 1.3 posterior to anterior joint space ratio at the central pole of the mandibular condyle, this patient is highly likely to have a disc displacement. In addition, if the patient has less than 1.7 superior to anterior joint space ratio at the lateral pole of the mandibular condyle, this patient is highly likely to have a disc displacement. Those patients need MRI evaluation for further assessment of disc position. When treating patients with a displaced disc, orthodontists need to be more cautious in securing the stable position of the disc and mandible before moving teeth to prevent unwanted relapse or TMD progression.

In the ROC analysis, the AUC and Youden’s index serve as an overall measure of diagnostic accuracy. Testing jointly on two approved values gives more reliable results than using a single index [21]. This study also provided separate optimal P-A and S-A cut-off ratios to identify possible disc-displaced joints to guide orthodontists when assessing the TMJ. When the P-A ratio is lower than 1.678 at the lateral pole or the S-A ratio is lower than 1.733 at the lateral pole, it has the best sensitivity and specificity to diagnose disc displacement based on ROC analysis.

The main limitation of this study is the lack of healthy TMJ subjects as controls. Instead, we used a control group that presented a normal joint ratio but had TMD symptoms. That might explain why the P-A and S-A ratio values in our control group are slightly lower than those in the previous study, which examined the healthy TMJ [13]. It is not ethical to take both CBCT and MRI on healthy patients without any TMD symptoms. Another limitation is that our cohort is all Caucasians, not reflecting the ethical variation. Therefore, future studies including diverse ethnicities can provide more evidence on the correlations between altered joint space and disc displacement.

## 5. Conclusions

There is a dramatic decrease in the P-A and S-A ratios in disc-displaced TMJs compared to the joints with optimal disc positions. CBCT images can serve as a diagnostic modality for disc displacement with high sensitivity and specificity. If the P-A ratio is less than 1.678 at the lateral pole or 1.339 at the central pole, or if the S-A ratio is less than 1.733 at the lateral pole or 1.916 at the medial pole of the mandibular condyle, the patient is highly likely to have disc displacement. The frequency of disc displacement and its huge effects on growth reinforce dentists and orthodontists to consider appropriate strategies for the early diagnosis and management of TMJ disc displacement.

## Figures and Tables

**Table 1:** The sample population based on the history and clinical examination according to the Diagnostic Criteria for Temporomandibular Disorders (DC/TMD).

	Myalgia	Local Myalgia	Myofascial Pain	Myofascial Pain with Referral	Arthralgia	Headache Attributed to TMD	Disc Displacement (DD) with Reduction	DD with Reduction with Intermittent Locking	DD without Reduction with Limited Opening	DD without Reduction without Limited Opening	Degenerative Joint Disease	Subluxation
Control	0	1	10	0	0	6	0	0	0	1	1	0
Experimental	0	6	27	2	0	6	8	15	5	3	12	0

**Table 2:** The number and percentage of disc displacement types in this study.

		N	Percentage		N	Percentage
Control group		14	21.2%			
Experimental group	Normal disc position (N)	7	10.6%			
	Disc Displacement (DD)	45	68.2%	Anterior-Medial	4	8.9%
				Anterior-only	11	24.4%
				Anterior-Lateral	30	66.7%
	Total	66	100.0%			

**Table 3:** The posterior to anterior joint space ratio in different disc position groups.

Posterior: Anterior Ratio		Lateral			Central			Medial			Total		
		Mean	S.D.	*p*	Mean	S.D.	*p*	Mean	S.D.	*p*	Mean	S.D.	*p*
**Control group**		1.55	0.28	<0.001 ^abcd^	1.42	0.30	<0.001 ^abcde^	1.39	0.32	<0.001 ^abdef^	1.46	0.21	<0.001 ^abcdef^
Experimental group	**Normal** **disc position**	1.06	0.48		0.91	0.21		1.01	0.13		0.99	0.23	
	Disc displacement	0.89	0.38		0.77	0.35		0.91	0.39		0.86	0.30	
	**Anterior-Medial**	0.89	0.25		0.64	0.11		0.64	0.06		0.72	0.08	
	**Anterior-only**	0.99	0.56		0.95	0.51		1.15	0.50		1.03	0.44	
	**Anterior-Lateral**	0.85	0.32		0.72	0.29		0.86	0.34		0.81	0.24	

*p*-values for one-way ANCOVA including age and sex in five different disc position groups (bold); ^a^: *p* < 0.05 when comparing the control group to the normal disc position group; ^b^: *p* < 0.05 when comparing the control group to the anterior-medial group; ^c^: *p* < 0.05 when comparing the control group to the anterior-only group; ^d^: *p* < 0.05 when comparing the control group to the anterior-lateral group; ^e^: *p* < 0.05 when comparing the anterior-only to the anterior-lateral group; ^f^: *p* < 0.05 when comparing the anterior-medial to the anterior-only group.

**Table 4:** The superior to anterior joint space ratio in different disc position groups.

Superior: Anterior Ratio		Lateral			Central			Medial			Total		
		Mean	S.D.	*p*	Mean	S.D.	*p*	Mean	S.D.	*p*	Mean	S.D.	*p*
**Control group**		1.77	0.29	<0.001 ^abcd^	1.87	0.44	<0.001 ^bcdf^	1.76	0.31	<0.001 ^bcdef^	1.80	0.27	<0.001 ^abcdf^
Experimental group	**Normal** **disc position**	1.29	0.47		1.44	0.31		1.59	0.45		1.44	0.33	
	Disc displacement	1.04	0.33		1.07	0.47		1.12	0.42		1.08	0.35	
	**Anterior-Medial**	0.98	0.25		1.13	0.72		1.07	0.44		1.06	0.46	
	**Anterior-only**	1.14	0.34		1.25	0.48		1.32	0.53		1.24	0.35	
	**Anterior-Lateral**	1.01	0.34		0.99	0.42		1.05	0.36		1.02	0.34	

*p*-values for one-way ANCOVA including age and sex in five different disc position groups (bold); ^a^: *p* < 0.05 when comparing the control group to the normal disc position group; ^b^: *p* < 0.05 when comparing the control group to the anterior-medial group; ^c^: *p* < 0.05 when comparing the control group to the anterior-only group; ^d^: *p* < 0.05 when comparing the control group to the anterior-lateral group; ^e^: when comparing the normal disc position group to the anterior-medial group; ^f^: when comparing the normal disc position group to the anterior-lateral group.

**Table 5:** Sensitivity, specificity, and Youden’s index for P-A ratio from lateral and central poles (Youden’s index ≥ 0.70).

	Cut-Off Point	Sensitivity	Specificity	Youden’s Index	AUC	95% CI
Lateral poles	1.678	0.911	0.929	0.840	0.94	0.87–1.00
	1.641	0.889	0.929	0.817		
	1.527	0.867	0.929	0.795		
	1.408	0.778	1.000	0.778		
	1.488	0.844	0.929	0.773		
	1.692	0.911	0.857	0.768		
	1.386	0.756	1.000	0.756		
	1.430	0.822	0.929	0.751		
	1.381	0.733	1.000	0.733		
	1.428	0.800	0.929	0.729		
	1.348	0.711	1.000	0.711		
	1.409	0.778	0.929	0.706		
Central poles	1.339	0.867	0.929	0.795	0.93	0.86–1.00
	1.467	0.933	0.857	0.790		
	1.224	0.844	0.929	0.773		
	1.452	0.911	0.857	0.768		
	1.216	0.822	0.929	0.751		
	1.434	0.889	0.857	0.746		
	1.572	0.956	0.786	0.741		
	1.209	0.800	0.929	0.729		
	1.374	0.867	0.857	0.724		
	1.476	0.933	0.756	0.719		
	1.186	0.778	0.929	0.706		

**Table 6:** Sensitivity, specificity and Youden’s index for S-A ratio from lateral and medial poles (Youden’s index ≥ 0.70).

	Cut-Off Point	Sensitivity	Specificity	Youden’s Index	AUC	95% CI
Lateral poles	1.733	0.911	0.929	0.840	0.94	0.88–1.00
	1.666	0.889	0.929	0.817		
	1.592	0.867	0.929	0.795		
	1.565	0.844	0.929	0.773		
	1.769	0.911	0.857	0.768		
	1.398	0.756	1.000	0.756		
	1.528	0.822	0.929	0.751		
	1.394	0.733	1.000	0.733		
	1.521	0.800	0.929	0.729		
	1.375	0.711	1.000	0.711		
	1.520	0.778	0.929	0.706		
Medial poles	1.916	0.822	0.929	0.751	0.91	0.84–0.99
	2.028	0.889	0.857	0.746		
	1.835	0.800	0.929	0.729		
	2.028	0.867	0.857	0.724		
	1.829	0.778	0.929	0.706		
	1.961	0.844	0.857	0.702		

**Table 7:** Sensitivity, specificity, and Youden’s index for total P-A and total S-A (Youden’s index ≥ 0.70).

	Cut-Off Point	Sensitivity	Specificity	Youden’s Index	AUC	95% CI
Total	1.294	0.844	1.000	0.844	0.96	0.90–1.00
P-A	1.235	0.822	1.000	0.822		
	1.357	0.889	0.929	0.817		
	1.508	0.956	0.857	0.813		
	1.182	0.800	1.000	0.800		
	1.316	0.867	0.929	0.795		
	1.436	0.933	0.857	0.790		
	1.180	0.778	1.000	0.778		
	1.305	0.844	0.929	0.773		
	1.427	0.911	0.857	0.768		
	1.167	0.756	1.000	0.756		
	1.382	0.889	0.857	0.746		
	1.512	0.956	0.786	0.741		
	1.144	0.733	1.000	0.733		
	1.129	0.711	1.000	0.711		
Total	1.492	0.800	1.000	0.800	0.93	0.86–0.99
S-A	1.769	0.867	0.929	0.795		
	1.461	0.778	1.000	0.778		
	1.707	0.844	0.929	0.773		
	1.899	0.911	0.857	0.768		
	1.367	0.756	1.000	0.756		
	1.653	0.822	0.929	0.751		
	1.832	0.889	0.857	0.746		
	1.361	0.733	1.000	0.733		
	1.571	0.800	0.929	0.729		
	1.814	0.867	0.857	0.724		
	1.353	0.711	1.000	0.711		

## Data Availability

The data presented in this study are available on request.

## Supplementary Materials

The following supporting information can be downloaded at: https://www.mdpi.com/article/10.3390/diagnostics14010044/s1.
