# Supplementary material for: Correlation between TMJ Space Alteration and Disc Displacement: A Retrospective CBCT and MRI Study
Source: Diagnostics (Basel). 2023 Dec 25;14(1):44. doi: 10.3390/diagnostics14010044 (PMC10802894; doi:10.3390/diagnostics14010044)
Supplement: Supplementary file 1 [file diagnostics-14-00044-s001.zip › diagnostics-2766425-supplementary.pdf]

Supplementary Table S1. Sensitivity analyses of the posterior-to-anterior joint space ratio in different disc position groups

Posterior: Anterior Ratio	Lateral			Central			Medial			Total		
	**Mean**	**S.D.**	***p***	**Mean**	**S.D.**	***p***	**Mean**	**S.D.**	***p***	**Mean**	**S.D.**	***p***
**66 joints of 37 patients (original results)**												
Control group	1.55	0.28		1.42	0.30		1.39	0.32		1.46	0.21	
Normal disc position	1.06	0.48	<0.001 *ab*	0.91	0.21	<0.001 *ab*	1.01	0.13	<0.001 *ab*	0.99	0.23	<0.001 *ab*
DD	0.89	0.38		0.77	0.35		0.91	0.39		0.86	0.30	
**Sensitivity analyses**												
**32 left joints of 32 patients**												
Control group	1.48	0.18		1.43	0.12		1.29	0.32		1.40	0.14	
Normal disc position	1.39	0.58	0.02 *b*	1.04	0.25	0.003 *b*	0.98	0.14	0.18	1.14	0.30	0.007 *b*
DD	0.93	0.45		0.82	0.41		0.96	0.45		0.91	0.36	
**34 right joints of 34 patients**												
Control group	1.62	0.35		1.42	0.43		1.49	0.31		1.51	0.26	
Normal disc position	0.82	0.21	<0.001 *ab*	0.80	0.13	<0.001 *ab*	1.03	0.14	<0.001 *ab*	0.88	0.08	<0.001 *ab*
DD	0.85	0.31		0.72	0.29		0.86	0.33		0.81	0.23	

*P-values for one-way ANCOVA including age and sex in three different disc position groups; *a*: p <0.05 when comparing control group to normal disc position group; *b*: p <0.05 when comparing control group to DD group; *c*: p <0.05 when comparing normal disc position to DD.

Supplementary Table S2. Sensitivity analyses of the superior to anterior joint space ratio in different disc position groups

Superior: Anterior Ratio	Lateral			Central			Medial			Total		
	**Mean**	**S.D.**	***p***	**Mean**	**S.D.**	***p***	**Mean**	**S.D.**	***p***	**Mean**	**S.D.**	***p***
**66 joints of 37 patients (original results)**												
Control group	1.77	0.29		1.87	0.44		1.76	0.31		1.80	0.27	
Normal disc position	1.29	0.47	<0.001 *ab*	1.44	0.31	<0.001 *b*	1.59	0.45	<0.001 *bc*	1.44	0.33	<0.001 *ab*
DD	1.04	0.33		1.07	0.47		1.12	0.42		1.08	0.35	
**Sensitivity analyses**												
**32 left joints of 32 patients**												
Control group	1.80	0.34		1.93	0.21		1.60	0.26		1.78	0.22	
Normal disc position	1.32	0.69	<0.001 *b*	1.37	0.30	<0.001 *b*	1.34	0.28	0.06	1.34	0.41	<0.001 *b*
DD	1.06	0.36		1.03	0.44		1.13	0.47		1.07	0.36	
**34 right joints of 34 patients**												
Control group	1.75	0.27		1.80	0.60		1.92	0.30		1.82	0.33	
Normal disc position	1.27	0.36	<0.001 *ab*	1.49	0.36	0.03 *b*	1.78	0.49	<0.001 *bc*	1.52	0.30	<0.001 *bc*
DD	1.02	0.31		1.11	0.50		1.11	0.38		1.08	0.36	

*P-values for one-way ANCOVA including age and sex in three different disc position groups; *a*: p <0.05 when comparing control group to normal disc position group; *b*: p <0.05 when comparing control group to DD group; *c*: p <0.05 when comparing normal disc position to DD.
